# (*E*)-3-[4-(Hex­yloxy)phen­yl]-1-(2-hydroxy­phen­yl)prop-2-en-1-one

**DOI:** 10.1107/S1600536809017577

**Published:** 2009-05-14

**Authors:** Zainab Ngaini, Siti Muhaini Haris Fadzillah, Hasnain Hussain, Ibrahim Abdul Razak, Hoong-Kun Fun

**Affiliations:** a Department of Chemistry, Faculty of Resource Science and Technology, Universiti Malaysia Sarawak, 94300 Kota Samarahan, Sarawak, Malaysia; b Department of Molecular Biology, Faculty of Resource Science and Technology, Universiti Malaysia Sarawak, 94300 Kota Samarahan, Sarawak, Malaysia; c X-ray Crystallography Unit, School of Physics, Universiti Sains Malaysia, 11800 USM, Penang, Malaysia

## Abstract

In the title compound, C_21_H_24_O_3_, the conformation of the enone group is *s*–*cis*. The benzene rings are inclined at an angle of 7.9 (1)°. The alk­oxy tail is planar, with a maximum deviation from the least-squares plane of 0.009 (2) Å, and adopts a *trans* conformation throughout. An intra­molecular O—H⋯O inter­action between the keto and hydr­oxy groups forms *S*(6) ring motifs. In the crystal, mol­ecules are arranged in a head-to-tail manner down the *a* axis and are subsequently stacked along the *b* axis, forming mol­ecular sheets parallel to the *ab* plane. The crystal structure is further stabilized by weak C—H⋯π inter­actions and short C⋯O [3.376 (2) Å] contacts.

## Related literature

For the biological properties of chalcone derivatives, see: Bhat *et al.* (2005 ▶); Xue *et al.* (2004 ▶); Zhao *et al.* (2005 ▶); Lee *et al.* (2006 ▶). For related structures, see: Razak, Fun, Ngaini, Rahman & Hussain (2009 ▶); Razak, Fun, Ngaini, Fadzillah & Hussain (2009*a* ▶,*b* ▶); Ngaini, Fadzillah *et al.* (2009 ▶); Ngaini, Rahman *et al.* (2009 ▶). For details of hydrogen-bond motifs, see: Bernstein *et al.* (1995 ▶). For the stability of the temperature controller used in the data collection, see: Cosier & Glazer (1986 ▶).
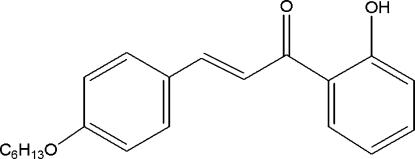


## Experimental

### 

#### Crystal data

C_21_H_24_O_3_
*M* *_r_* = 324.40
Monoclinic, 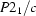

*a* = 19.6443 (5) Å
*b* = 7.1966 (2) Å
*c* = 12.6520 (3) Å
β = 106.438 (2)°
*V* = 1715.53 (8) Å^3^
*Z* = 4
Mo *K*α radiation
μ = 0.08 mm^−1^
*T* = 100 K
0.47 × 0.12 × 0.04 mm

#### Data collection

Bruker SMART APEXII CCD area-detector diffractometer
Absorption correction: multi-scan (*SADABS*; Bruker, 2005 ▶) *T*_min_ = 0.962, *T*_max_ = 0.997
20873 measured reflections
5025 independent reflections
2783 reflections with *I* > 2σ(*I*)
*R*_int_ = 0.057

#### Refinement

*R*[*F* ^2^ > 2σ(*F* ^2^)] = 0.070
*wR*(*F* ^2^) = 0.190
*S* = 1.05
5025 reflections
222 parameters
H atoms treated by a mixture of independent and constrained refinement
Δρ_max_ = 0.53 e Å^−3^
Δρ_min_ = −0.29 e Å^−3^

### 

Data collection: *APEX2* (Bruker, 2005 ▶); cell refinement: *SAINT* (Bruker, 2005 ▶); data reduction: *SAINT*; program(s) used to solve structure: *SHELXTL* (Sheldrick, 2008 ▶); program(s) used to refine structure: *SHELXTL*; molecular graphics: *SHELXTL*; software used to prepare material for publication: *SHELXTL* and *PLATON* (Spek, 2009 ▶).

## Supplementary Material

Crystal structure: contains datablocks global, I. DOI: 10.1107/S1600536809017577/sj2623sup1.cif

Structure factors: contains datablocks I. DOI: 10.1107/S1600536809017577/sj2623Isup2.hkl

Additional supplementary materials:  crystallographic information; 3D view; checkCIF report

## Figures and Tables

**Table 1:** Hydrogen-bond geometry (Å, °)

*D*—H⋯*A*	*D*—H	H⋯*A*	*D*⋯*A*	*D*—H⋯*A*
O1—H1*O*1⋯O2	0.90 (3)	1.68 (3)	2.507 (2)	152 (2)
C20—H20*A*⋯*Cg*1^i^	0.97	2.84	3.657 (2)	142
C20—H20*B*⋯*Cg*1^ii^	0.97	2.78	3.637 (2)	147

Symmetry codes: (i) 
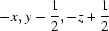
; (ii) 
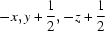
. *Cg*1 is the centroid of the C1-C6 ring.

**Fig. 1.**
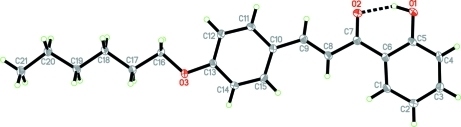
The molecular structure of the title compound, showing 50% probability displacement ellipsoids and the atom numbering scheme. The intramolecular interaction is shown as dashed line.

**Fig. 2.**
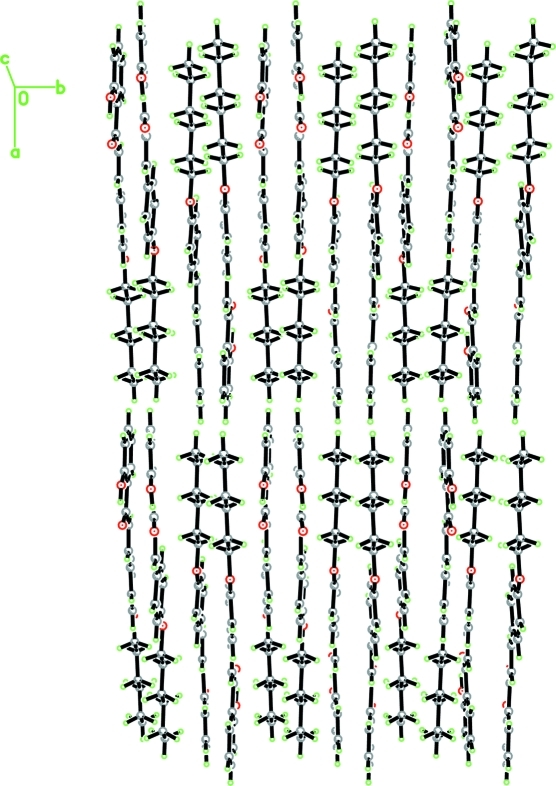
The crystal packing of the title compound, viewed down the c axis.

## supplementary crystallographic information

### Comment

The biological properties of chalcones derivatives, such as their anticancer (Bhat *et al.*, 2005), antimalarial (Xue *et al.*, 2004), antiangiogenic and antitumour (Lee *et al.*, 2006) and antiplatelet (Zhao *et al.*, 2005) activities, have been extensively reported. Synthetic and naturally occurring chalcones are of interest and have been widely studied and developed as one of the pharmaceutically important molecules. As part of our studies, we have synthesized the title chalcone derivative, (I). Its antibacterial activity was tested against *E. coli* ATCC 8739 and the compound demonstrated antimicrobial activity. In this paper, we report the crystal structure of the title compound.

The bond lengths observed in the title compound (Fig.1) are comparable with those reported by Allen *et al.* (1987). The enone (O2/C7—C9) moiety adopts *s*-*cis* conformation with a O2—C7—C8—C9 torsion angle of -3.6 (3)°. The mean plane through the enone moiety makes dihedral angles of 0.89 (1)° and 7.9 (1)° with the C1—C6 and C10—C15 benzene rings, respectively. The dihedral angle between the two benzene rings is 7.9 (1)°.

The slight opening of the C1—C6—C7 (123.4 (2)°) and C6—C7—C8 (121.1 (2)°) angles is the result of the short H1A···H8A (2.15 Å) contact whereas close interatomic contact between H8A and H15A (2.36 Å) widened the C8—C9—C10 and C9—C10—C15 angles to 129.2 (2)° and 124.1 (2)°, respectively. Likewise, strain induced by short H12A···H16A (2.39 Å) and H12A···H16B (2.33 Å) contacts resulted in the opening of the O3—C13—C12 (125.1 (2)°) angle. Similar features were also reported in related structures (Razak, Fun, Ngaini, Rahman *et al.*, 2009; Razak, Fun, Ngaini, Fadzillah & Hussain, 2009*a*,*b*; Ngaini, Fadzillah *et al.*, 2009; Ngaini, Rahman *et al.*, 2009).

The zigzag alkoxyl tail adopts an all-*trans* conformation with the largest deviation from the ideal value being -179.3 (2)° for C17—C18—C19—C20 torsion angle. The alkoxyl chain is planar with the maximum deviation from the least-squares plane of 0.009 (2)Å at C18. The zigzag plane makes a dihedral angle of 2.2 (1)° with the attached benzene ring.

The keto and hydroxy groups in the molecule form an intramolecular O1—H1O1···O2 interaction (Table 1) generating a ring of graph-set motif *S*(6) (Bernstein *et al.*, 1995). In the crystal structure, the molecules are arranged into a head-to-tail manner down the *a* axis (Fig. 2). Molecules are  subsequently stacked along the *b* axis, forming molecular sheets parallel to the *ab* plane. In the absence of conventional hydrogen bonds, the crystal packing is strengthened by the presence of weak C—H···π interactions between atom C20 of the alkoxyl tail and the C1—C6 benzene ring (Table 1).  There is also a short C···O (x, 1.5-y, 0.5+z )  [3.376 (2)Å] contact.

### Experimental

A mixture of 2-hydroxyacetophenone (2.72 ml, 20 mmol) and 4-hexyloxybenzaldehyde (4.12 ml, 20 mmol) and KOH (4.04 g, 72 mmol) in 60 ml of methanol was heated at reflux for 24 h. The reaction was cooled to room temperature and acidified with cold diluted HCl (2 M). The resulting precipitate was filtered, washed and dried. After redissolving in hexane, followed by few days of slow evaporation, crystals were collected.

### Refinement

The O-bound H atom was located in a difference Fourier map and refined freely. All the C-bound H atoms were positioned geometrically and refined using a riding model with C—H = 0.93–0.97 Å. The *U*_iso_ values were constrained to be -1.5U_equ_ (methyl H atoms) and -1.2U_equ_ (other H atoms). The rotating model group was applied for the methyl group.

### Crystal data

**Table:**

C_21_H_24_O_3_	*F*(000) = 696
*M**_r_* = 324.40	*D*_x_ = 1.256 Mg m^−^^3^
Monoclinic, *P*2_1_/*c*	Mo *K*α radiation, λ = 0.71073 Å
Hall symbol: -P 2ybc	Cell parameters from 3217 reflections
*a* = 19.6443 (5) Å	θ = 3.0–30.0°
*b* = 7.1966 (2) Å	µ = 0.08 mm^−^^1^
*c* = 12.6520 (3) Å	*T* = 100 K
β = 106.438 (2)°	Needle, yellow
*V* = 1715.53 (8) Å^3^	0.47 × 0.12 × 0.04 mm
*Z* = 4	

### Data collection

**Table:**

Bruker SMART APEXII CCD area-detector diffractometer	5025 independent reflections
Radiation source: sealed tube	2783 reflections with *I* > 2σ(*I*)
graphite	*R*_int_ = 0.057
φ and ω scans	θ_max_ = 30.1°, θ_min_ = 1.1°
Absorption correction: multi-scan (*SADABS*; Bruker, 2005)	*h* = −27→27
*T*_min_ = 0.962, *T*_max_ = 0.997	*k* = −9→10
20873 measured reflections	*l* = −17→17

### Refinement

**Table:**

Refinement on *F*^2^	Primary atom site location: structure-invariant direct methods
Least-squares matrix: full	Secondary atom site location: difference Fourier map
*R*[*F*^2^ > 2σ(*F*^2^)] = 0.070	Hydrogen site location: inferred from neighbouring sites
*wR*(*F*^2^) = 0.190	H atoms treated by a mixture of independent and constrained refinement
*S* = 1.05	*w* = 1/[σ^2^(*F*_o_^2^) + (0.0907*P*)^2^] where *P* = (*F*_o_^2^ + 2*F*_c_^2^)/3
5025 reflections	(Δ/σ)_max_ < 0.001
222 parameters	Δρ_max_ = 0.53 e Å^−^^3^
0 restraints	Δρ_min_ = −0.29 e Å^−^^3^

### Special details

**Table:**

Experimental. The crystal was placed in the cold stream of an Oxford Cryosystems Cobra open-flow nitrogen cryostat (Cosier & Glazer, 1986) operating at 100.0 (1) K.
Geometry. All e.s.d.'s (except the e.s.d. in the dihedral angle between two l.s. planes) are estimated using the full covariance matrix. The cell e.s.d.'s are taken into account individually in the estimation of e.s.d.'s in distances, angles and torsion angles; correlations between e.s.d.'s in cell parameters are only used when they are defined by crystal symmetry. An approximate (isotropic) treatment of cell e.s.d.'s is used for estimating e.s.d.'s involving l.s. planes.
Refinement. Refinement of *F*^2^ against ALL reflections. The weighted *R*-factor *wR* and goodness of fit *S* are based on *F*^2^, conventional *R*-factors *R* are based on *F*, with *F* set to zero for negative *F*^2^. The threshold expression of *F*^2^ > σ(*F*^2^) is used only for calculating *R*-factors(gt) *etc*. and is not relevant to the choice of reflections for refinement. *R*-factors based on *F*^2^ are statistically about twice as large as those based on *F*, and *R*- factors based on ALL data will be even larger.

### Fractional atomic coordinates and isotropic or equivalent isotropic displacement parameters (Å^2^)

**Table:**

	*x*	*y*	*z*	*U*_iso_*/*U*_eq_	
O1	0.31582 (7)	0.61343 (19)	−0.09249 (11)	0.0210 (3)	
O2	0.20488 (6)	0.60487 (18)	−0.03200 (11)	0.0203 (3)	
O3	−0.06969 (6)	0.61140 (17)	0.36275 (11)	0.0168 (3)	
C1	0.35907 (9)	0.6404 (2)	0.20871 (16)	0.0159 (4)	
H1A	0.3371	0.6462	0.2648	0.019*	
C2	0.43212 (9)	0.6445 (3)	0.23554 (17)	0.0202 (4)	
H2A	0.4590	0.6512	0.3089	0.024*	
C3	0.46535 (9)	0.6386 (3)	0.15149 (16)	0.0194 (4)	
H3A	0.5146	0.6415	0.1691	0.023*	
C4	0.42582 (9)	0.6283 (3)	0.04267 (16)	0.0175 (4)	
H4A	0.4485	0.6249	−0.0126	0.021*	
C5	0.35182 (9)	0.6230 (2)	0.01518 (15)	0.0149 (4)	
C6	0.31698 (9)	0.6276 (2)	0.09933 (15)	0.0137 (4)	
C7	0.23827 (9)	0.6181 (2)	0.06736 (15)	0.0138 (4)	
C8	0.19935 (9)	0.6221 (2)	0.15096 (16)	0.0146 (4)	
H8A	0.2236	0.6250	0.2256	0.018*	
C9	0.12783 (9)	0.6216 (2)	0.11734 (16)	0.0153 (4)	
H9A	0.1076	0.6215	0.0414	0.018*	
C10	0.07792 (8)	0.6213 (2)	0.18274 (15)	0.0130 (4)	
C11	0.00511 (9)	0.6329 (2)	0.12710 (16)	0.0154 (4)	
H11A	−0.0091	0.6438	0.0507	0.018*	
C12	−0.04606 (9)	0.6284 (3)	0.18360 (16)	0.0160 (4)	
H12A	−0.0940	0.6344	0.1454	0.019*	
C13	−0.02488 (9)	0.6148 (2)	0.29733 (16)	0.0135 (4)	
C14	0.04731 (9)	0.6028 (2)	0.35469 (16)	0.0156 (4)	
H14A	0.0612	0.5925	0.4311	0.019*	
C15	0.09760 (9)	0.6064 (2)	0.29794 (16)	0.0154 (4)	
H15A	0.1454	0.5988	0.3366	0.019*	
C16	−0.14479 (8)	0.6211 (3)	0.31008 (15)	0.0153 (4)	
H16A	−0.1602	0.5148	0.2621	0.018*	
H16B	−0.1565	0.7335	0.2664	0.018*	
C17	−0.18025 (9)	0.6212 (3)	0.40186 (15)	0.0151 (4)	
H17A	−0.1631	0.7269	0.4496	0.018*	
H17B	−0.1667	0.5094	0.4455	0.018*	
C18	−0.26094 (9)	0.6306 (3)	0.36018 (15)	0.0149 (4)	
H18A	−0.2785	0.5255	0.3122	0.018*	
H18B	−0.2750	0.7434	0.3176	0.018*	
C19	−0.29383 (9)	0.6284 (3)	0.45638 (15)	0.0144 (4)	
H19A	−0.2785	0.5166	0.4994	0.017*	
H19B	−0.2759	0.7341	0.5037	0.017*	
C20	−0.37459 (9)	0.6351 (3)	0.42130 (16)	0.0162 (4)	
H20A	−0.3931	0.5290	0.3747	0.019*	
H20B	−0.3904	0.7469	0.3786	0.019*	
C21	−0.40379 (9)	0.6331 (3)	0.52132 (17)	0.0219 (5)	
H21A	−0.4547	0.6385	0.4969	0.033*	
H21B	−0.3859	0.7386	0.5672	0.033*	
H21C	−0.3892	0.5210	0.5626	0.033*	
H1O1	0.2698 (14)	0.605 (3)	−0.094 (2)	0.059 (9)*	

### Atomic displacement parameters (Å^2^)

**Table:**

	*U*^11^	*U*^22^	*U*^33^	*U*^12^	*U*^13^	*U*^23^
O1	0.0199 (7)	0.0333 (9)	0.0108 (7)	−0.0030 (6)	0.0060 (6)	0.0011 (6)
O2	0.0166 (6)	0.0309 (8)	0.0131 (7)	0.0007 (6)	0.0035 (6)	0.0021 (6)
O3	0.0115 (6)	0.0264 (8)	0.0139 (7)	0.0003 (5)	0.0060 (5)	−0.0003 (6)
C1	0.0167 (8)	0.0195 (10)	0.0137 (10)	0.0012 (8)	0.0078 (7)	0.0006 (8)
C2	0.0174 (9)	0.0295 (12)	0.0132 (10)	−0.0017 (8)	0.0035 (8)	0.0002 (9)
C3	0.0142 (8)	0.0238 (11)	0.0213 (11)	−0.0006 (8)	0.0068 (8)	0.0018 (9)
C4	0.0179 (8)	0.0193 (10)	0.0198 (11)	0.0006 (8)	0.0125 (8)	0.0021 (9)
C5	0.0203 (9)	0.0132 (10)	0.0127 (10)	−0.0009 (7)	0.0071 (8)	0.0018 (8)
C6	0.0149 (8)	0.0130 (10)	0.0146 (10)	0.0012 (7)	0.0068 (7)	0.0004 (8)
C7	0.0154 (8)	0.0125 (9)	0.0147 (10)	0.0010 (7)	0.0061 (7)	0.0016 (8)
C8	0.0149 (8)	0.0162 (10)	0.0135 (10)	0.0005 (7)	0.0055 (7)	0.0018 (8)
C9	0.0164 (8)	0.0146 (10)	0.0157 (10)	0.0012 (7)	0.0061 (7)	0.0005 (8)
C10	0.0122 (8)	0.0126 (9)	0.0149 (10)	−0.0001 (7)	0.0049 (7)	−0.0004 (8)
C11	0.0162 (8)	0.0180 (10)	0.0119 (10)	0.0012 (7)	0.0041 (7)	0.0008 (8)
C12	0.0120 (8)	0.0196 (10)	0.0168 (10)	0.0023 (7)	0.0046 (7)	0.0017 (9)
C13	0.0135 (8)	0.0132 (9)	0.0152 (10)	0.0000 (7)	0.0062 (7)	−0.0021 (8)
C14	0.0152 (8)	0.0202 (11)	0.0111 (10)	−0.0004 (7)	0.0031 (7)	0.0007 (8)
C15	0.0115 (8)	0.0173 (10)	0.0170 (10)	−0.0010 (7)	0.0033 (7)	−0.0027 (8)
C16	0.0102 (7)	0.0197 (10)	0.0156 (10)	0.0000 (7)	0.0030 (7)	−0.0002 (8)
C17	0.0145 (8)	0.0177 (10)	0.0142 (10)	0.0016 (7)	0.0061 (7)	0.0004 (8)
C18	0.0148 (8)	0.0167 (10)	0.0153 (10)	−0.0005 (7)	0.0076 (7)	−0.0008 (8)
C19	0.0148 (8)	0.0158 (10)	0.0138 (10)	0.0000 (7)	0.0060 (7)	0.0003 (8)
C20	0.0150 (8)	0.0172 (10)	0.0175 (10)	−0.0002 (7)	0.0065 (7)	0.0011 (8)
C21	0.0176 (9)	0.0283 (12)	0.0231 (12)	0.0018 (8)	0.0112 (8)	0.0040 (10)

### Geometric parameters (Å, °)

**Table:**

O1—C5	1.347 (2)	C12—C13	1.384 (3)
O1—H1O1	0.90 (3)	C12—H12A	0.9300
O2—C7	1.246 (2)	C13—C14	1.401 (2)
O3—C13	1.369 (2)	C14—C15	1.376 (2)
O3—C16	1.438 (2)	C14—H14A	0.9300
C1—C2	1.378 (2)	C15—H15A	0.9300
C1—C6	1.400 (3)	C16—C17	1.514 (2)
C1—H1A	0.9300	C16—H16A	0.9700
C2—C3	1.397 (3)	C16—H16B	0.9700
C2—H2A	0.9300	C17—C18	1.524 (2)
C3—C4	1.378 (3)	C17—H17A	0.9700
C3—H3A	0.9300	C17—H17B	0.9700
C4—C5	1.396 (2)	C18—C19	1.531 (2)
C4—H4A	0.9300	C18—H18A	0.9700
C5—C6	1.420 (2)	C18—H18B	0.9700
C6—C7	1.485 (2)	C19—C20	1.522 (2)
C7—C8	1.470 (2)	C19—H19A	0.9700
C8—C9	1.348 (2)	C19—H19B	0.9700
C8—H8A	0.9300	C20—C21	1.530 (3)
C9—C10	1.451 (2)	C20—H20A	0.9700
C9—H9A	0.9300	C20—H20B	0.9700
C10—C15	1.402 (3)	C21—H21A	0.9600
C10—C11	1.406 (2)	C21—H21B	0.9600
C11—C12	1.389 (2)	C21—H21C	0.9600
C11—H11A	0.9300		
			
C5—O1—H1O1	105.5 (18)	C15—C14—H14A	120.0
C13—O3—C16	118.03 (14)	C13—C14—H14A	120.0
C2—C1—C6	121.83 (17)	C14—C15—C10	121.08 (16)
C2—C1—H1A	119.1	C14—C15—H15A	119.5
C6—C1—H1A	119.1	C10—C15—H15A	119.5
C1—C2—C3	119.35 (19)	O3—C16—C17	106.20 (15)
C1—C2—H2A	120.3	O3—C16—H16A	110.5
C3—C2—H2A	120.3	C17—C16—H16A	110.5
C4—C3—C2	120.63 (17)	O3—C16—H16B	110.5
C4—C3—H3A	119.7	C17—C16—H16B	110.5
C2—C3—H3A	119.7	H16A—C16—H16B	108.7
C3—C4—C5	120.20 (17)	C16—C17—C18	113.20 (15)
C3—C4—H4A	119.9	C16—C17—H17A	108.9
C5—C4—H4A	119.9	C18—C17—H17A	108.9
O1—C5—C4	117.70 (16)	C16—C17—H17B	108.9
O1—C5—C6	122.21 (16)	C18—C17—H17B	108.9
C4—C5—C6	120.09 (17)	H17A—C17—H17B	107.8
C1—C6—C5	117.89 (15)	C17—C18—C19	110.86 (15)
C1—C6—C7	123.40 (16)	C17—C18—H18A	109.5
C5—C6—C7	118.71 (17)	C19—C18—H18A	109.5
O2—C7—C8	119.66 (16)	C17—C18—H18B	109.5
O2—C7—C6	119.24 (15)	C19—C18—H18B	109.5
C8—C7—C6	121.09 (17)	H18A—C18—H18B	108.1
C9—C8—C7	118.73 (18)	C20—C19—C18	114.03 (15)
C9—C8—H8A	120.6	C20—C19—H19A	108.7
C7—C8—H8A	120.6	C18—C19—H19A	108.7
C8—C9—C10	129.22 (19)	C20—C19—H19B	108.7
C8—C9—H9A	115.4	C18—C19—H19B	108.7
C10—C9—H9A	115.4	H19A—C19—H19B	107.6
C15—C10—C11	117.84 (15)	C19—C20—C21	111.23 (16)
C15—C10—C9	124.12 (16)	C19—C20—H20A	109.4
C11—C10—C9	118.03 (17)	C21—C20—H20A	109.4
C12—C11—C10	121.51 (17)	C19—C20—H20B	109.4
C12—C11—H11A	119.2	C21—C20—H20B	109.4
C10—C11—H11A	119.2	H20A—C20—H20B	108.0
C13—C12—C11	119.23 (16)	C20—C21—H21A	109.5
C13—C12—H12A	120.4	C20—C21—H21B	109.5
C11—C12—H12A	120.4	H21A—C21—H21B	109.5
O3—C13—C12	125.07 (15)	C20—C21—H21C	109.5
O3—C13—C14	114.56 (16)	H21A—C21—H21C	109.5
C12—C13—C14	120.37 (16)	H21B—C21—H21C	109.5
C15—C14—C13	119.97 (17)		
			
C6—C1—C2—C3	0.9 (3)	C8—C9—C10—C11	175.97 (18)
C1—C2—C3—C4	−0.1 (3)	C15—C10—C11—C12	−0.5 (3)
C2—C3—C4—C5	−0.2 (3)	C9—C10—C11—C12	178.26 (17)
C3—C4—C5—O1	179.86 (18)	C10—C11—C12—C13	0.9 (3)
C3—C4—C5—C6	−0.2 (3)	C16—O3—C13—C12	0.5 (2)
C2—C1—C6—C5	−1.3 (3)	C16—O3—C13—C14	−179.34 (15)
C2—C1—C6—C7	178.43 (16)	C11—C12—C13—O3	179.13 (17)
O1—C5—C6—C1	−179.10 (17)	C11—C12—C13—C14	−1.0 (3)
C4—C5—C6—C1	1.0 (3)	O3—C13—C14—C15	−179.48 (16)
O1—C5—C6—C7	1.1 (3)	C12—C13—C14—C15	0.6 (3)
C4—C5—C6—C7	−178.82 (16)	C13—C14—C15—C10	−0.2 (3)
C1—C6—C7—O2	−179.21 (18)	C11—C10—C15—C14	0.1 (3)
C5—C6—C7—O2	0.6 (3)	C9—C10—C15—C14	−178.55 (17)
C1—C6—C7—C8	0.2 (3)	C13—O3—C16—C17	−178.12 (14)
C5—C6—C7—C8	179.95 (16)	O3—C16—C17—C18	−179.87 (14)
O2—C7—C8—C9	−3.6 (3)	C16—C17—C18—C19	179.43 (15)
C6—C7—C8—C9	177.02 (16)	C17—C18—C19—C20	−179.30 (15)
C7—C8—C9—C10	178.62 (18)	C18—C19—C20—C21	−179.83 (15)
C8—C9—C10—C15	−5.4 (3)		

### Hydrogen-bond geometry (Å, °)

**Table:**

*D*—H···*A*	*D*—H	H···*A*	*D*···*A*	*D*—H···*A*
O1—H1O1···O2	0.90 (3)	1.68 (3)	2.507 (2)	152 (2)
C20—H20A···Cg1^i^	0.97	2.84	3.657 (2)	142
C20—H20B···Cg1^ii^	0.97	2.78	3.637 (2)	147

Symmetry codes: (i) −*x*, *y*−1/2, −*z*+1/2; (ii) −*x*, *y*+1/2, −*z*+1/2.
